# Low prevalence of scabies and impetigo in Dakar/Senegal: A cluster-randomised, cross-sectional survey

**DOI:** 10.1371/journal.pgph.0002942

**Published:** 2024-02-28

**Authors:** Andreas Hansmann, Genevia Wamba Lékémo, Chiaka Fomba, Jade Kaddoura, Ramatoullaye Toure, Assane Diop, Maodo Ndiaye, Olivier Chosidow, Michael Marks, Fatimata Ly

**Affiliations:** 1 Department of Neonatology and Paediatric Intensive Care Medicine, University Hospital Bonn, Bonn, Germany; 2 Service de Dermatologie, Institut d’Hygiène Sociale, Université Cheikh Anta Diop, Dakar, Sénégal; 3 Hospital Aristide Le Dantec, Dakar, Sénégal; 4 Service de Dermatologie, Henri Mondor Hospital, Université Paris-Est, Créteil, Paris, France; 5 Department of Clinical Research, London School of Hygiene and Tropical Medicine, London, United Kingdom; University of Colorado Anschutz Medical Campus: University of Colorado - Anschutz Medical Campus, UNITED STATES

## Abstract

**Background:**

Scabies, a parasitic infection caused by *Sarcoptes scabiei var*. *hominis*, *is* a public health problem with significant morbidity worldwide, particularly in low-resource countries. Impetigo, a complication of scabies infection, is a risk factor for sepsis, glomerulonephritis and possibly acute rheumatic fever. Currently, the majority of epidemiological data has been collected in rural populations in the Pacific with limited applicability to urban populations in sub-Saharan Africa, where scabies is also believed to be a problem. To inform future public health programs, more reliable information about the burden of disease is required.

**Methodology/Principal findings:**

In July/August 2022, we conducted a cross sectional, cluster-randomised, household survey in Pikine/Dakar using the ‘International Association for the Control of Scabies (IACS)’ criteria to diagnose scabies and impetigo. All participants underwent a standardised clinical examination by post-graduate dermatology students. For those diagnosed with scabies, an age-adapted ‘Dermatology Life Quality Index (DLQI)’ questionnaire was filled. We recruited and examined 1697 participants to detect 27 cases of scabies (prevalence: 1.6%, 95% CI 0.8–3.2), mostly in school aged children. Ten participants suffered from impetigo (prevalence: 0.6%, 95% CI 0.3–1.3), 5 of which were dually infected with scabies. Risk factors for scabies infection were young age, male gender and Koranic school attendance. Of those found to have scabies, in 7 out of 22 cases (31.8%) it had a large effect on their lives according to the DLQI questionnaires filled.

**Conclusions/Significance:**

This study adds to the mapping of the burden of scabies across Africa to support public health action. With a low prevalence of scabies that is concentrated amongst poor households and children attending Koranic schools, a focused public health approach targeting Koranic schools and poor households seems to be most appropriate in this community.

## 1. Introduction

Scabies is a parasitic skin infection caused by the *Sarcoptes scabiei var*. *hominis* mite. It is estimated that worldwide more than 200 million people live with scabies and 455 million new cases occur every year, accounting for 4.5 million DALYs [1–3]. Whilst scabies is endemic worldwide, the largest burden is recorded in low-income, tropical countries with children and the poorest in society disproportionally affected [4,5].

Scabies is transmitted predominantly through close skin-to-skin contact within households or in institutional settings [6]. Female mites burrow into the skin of the human host to deposit eggs and excrement [7]. Intense itch occurs from three weeks after infection, leading to distinctive skin eruptions. The intense itch, scratching and skin changes can lead to psychosocial consequences including shame, stigma, isolation, sleep disturbance and absenteeism from school or work [8].

Scabies can also have serious health outcomes via bacterial superinfection (‘impetiginisation of scabies’) with *Streptococcus pyogenes* or *Staphylococcus aureus*. This can contribute significantly to the burden of local and systemic bacterial infection, including necrotizing soft tissue infection, sepsis, post-streptococcal glomerulonephritis [9–12] and possibly rheumatic fever and rheumatic heart disease [13,14]. Because scabies is so common and can cause potentially severe health outcomes through impetiginisation, there has been renewed effort to control the disease.

In 2017 WHO added scabies to its list of Neglected Tropical Diseases (NTDs) and noted that mapping of the disease’s prevalence needs to occur before large-scale activities associated with scabies prevention and control can begin [15]. To identify communities who would benefit from this intervention, WHO advocates for a rapid mapping of scabies prevalence in suspected high-burden countries [16]. To standardize efforts, the International Alliance for the Control of Scabies (IACS) published consensus criteria for the diagnosis of scabies in 2018 [17,18]. Initial surveys have demonstrated the usefulness and reliability of these criteria [19–21].

Geographically, most population prevalence surveys have been conducted in the Pacific and have frequently recorded very high prevalence rates amongst the general populations [22–25]. In Africa, a community-wide survey in rural Malawi found scabies and impetigo prevalence rates of 15% and 7% respectively [26]. In the Amhara Region of Ethiopia, a population-based survey amongst children aged 5 to 14 years reported a scabies prevalence of 11% [27]. In peri-urban Monrovia a population-based survey of scabies and impetigo recorded a scabies-prevalence of 9%, half of which was severe (52%). 1% of the survey population suffered from impetigo [21]. Surveys in Guinea Bissau and the Gambia have found a prevalence of scabies of 5 and 15% respectively amongst children [28,29].

In Senegal, to our knowledge, no large population-based surveys of scabies or impetigo has been conducted in the past, though anecdotal evidence suggests a high burden of disease. A recent survey of 15 Koranic schools, called ‘daaras’ in Dakar, found a prevalence of 7.3% amongst pupils, with a range from 2 to 31% between individual institutions [30]. Daaras are residential schools where students learn the Koran, at times in very crowded circumstances. Surveys in prisons and dermatologists practicing in public hospitals in Dakar confirm a high burden of scabies in their out-patient clinics (Ly, personal communication). We therefore undertook a cluster-randomised, cross-sectional survey employing a standardised diagnostic approach to diagnose scabies and impetigo in peri-urban Dakar.

## 2. Methods

The survey took place in Pikine, a neighbourhood of about 2 km x 3 km in size that lies 10 km outside the centre of Dakar, the capital of Senegal. Pikine is a very densely populated area of single and multi-story concrete and cement brick houses, arranged into a square grid by sand and tarmac streets. The mostly Wolof-speaking, multi-ethnic population is a mix of long-term residents and more recent arrivals. It is considered one of the poorer parts of Dakar but public services such as sanitation, waste removal and piped water are available to most inhabitants.

The border of Pikine was mapped using Google maps. Each block of houses was numbered (530 blocks in total). We excluded 24 blocks of houses containing only large commercial or public buildings (17 blocks) or those, who were too large to be enlisted in a single day by the study team (7 blocks). We then randomly selected 27 housing blocks using simple random selection. The cluster size (= number of people living in one block) was selected to allow the study team to be able to visit all households and enlist all household members within the cluster in one full working day.

Two final year post-graduate dermatology students and two second-year post-graduate dermatology students from Université Cheikh Anta Diop Dakar were trained during a 2-day practical training course to diagnose scabies according to the IACS criteria [18]. Community health volunteers accompanied the team and helped with translation where this was necessary.

During the household visit, basic demographic and socio-economic data and a standardised history was collected on all participants, who were also examined for scabies and impetigo. The examination involved only the arms up to the axilla, exposed parts of the legs and the face. This has been shown to diagnose 90% of all scabies cases compared to a full body exam in a validation trial [31]. Health-related quality of life data was collected from individuals with scabies. All data was collected using tablets and ODK (Open Data Kit) software.

A case of scabies was defined as fulfilling the 2020 IACS’s criteria of either clinical scabies or suspected scabies [18]. Dermatoscopy or other methods of parasite identification were not available in this community trial to confirm the diagnosis in our participants. The severity of the presentation was classified by the number of lesions present as mild (≤10 lesions), moderate (11–49 lesions) or severe (≥ 50 lesions or crusted scabies) [31]. Impetigo was diagnosed on the basis of papular, pustular or ulcerative lesions with associated erythema, crusting, bullae or pus. Severity of impetigo was classified as very mild (≤ 5 lesions), mild (6–10 lesions), moderate (11–49 lesions) or severe (≥ 50 lesions) as described before [31].

All patients found to have scabies or impetigo were provided treatment free of charge according to local standard treatment guidelines. Scabies was treated with weight based oral ivermectin on day 0 of diagnosis and day 7, or topical benzyl benzoate 10% lotion for children with a weight of less than 15 kg. Impetigo was treated with oral antibiotics for 5 days and antiseptic wound care. Symptomatic household contacts were also treated. Other moderate or severe dermatological conditions were referred for treatment.

Health-related quality of life (HRQOL) was assessed using the Dermatology Quality of Life Index (DLQI) for those 16 years of age or older, the Children’s DLQI (CDLQI) for children aged 7 to 15 years and the Family DLQI (FDLQI) for children 6 years or below. The DLQIs have been used previously for scabies [32], are validated for use on tablets [33], and validated translations into French are available [34]. Where French was not the mother tongue of the participant, the questions were read out and translated from French into Wolof by the fieldworkers or the community health workers.

Sample size calculation: We assumed that with a design-effect of 1.2 and an average cluster size of 100, we needed to enrol 1600 individuals to detect a prevalence of scabies of 10% with 2% precision. Assuming an average household size of 5, we planned to enrol 16 clusters of 20 households. As the average cluster size was less than 100 participants, we increased the number of randomly selected clusters to 27 to meet our target of 1600 participants.

We calculated the prevalence of scabies and impetigo stratified by demographic variables. For children age was grouped in steps of 5-years until the age of twenty. The remaining adult population was summarised in a single age category. The education of the head of the household was categorised as either no schooling, schooling up to partial secondary school education or at least secondary school education. The type of school was categorised as either Koranic school or any other school. Household size was defined as how many people slept in the house the previous night. The svyset STATA command was used to adjust for the cluster randomized sampling design. For proportions, robust standard errors were used to calculate confidence intervals adjusted for clustering at the community level. Logistic regression analysis was done using random-effects regression adjusted for clustering as above. Due to the small number of outcomes, univariate modelling was chosen for key socio-demographic variables to avoid overfitting of the model [35]. The relationship between HRQOL and scabies was assessed by calculating the FDLQI, CDLQI and DLQI according to age. Median scores and inter-quartile ranges (IQR) were used to quantify the effect on the QOL. The impact of scabies on the quality of life was assessed as no effect on patient’s life (DLQI scores 0–1), small effect on patient’s life (2–5), moderate effect on patient’s life (6–10), very large effect on patient’s life (11–20) and extremely large effect on patient’s life (21–30) [36]. Stata SE 17.0 (StataCorp, College Station, TX) was used for data analysis.

The study was approved by the London School of Hygiene and Tropical Medicine MSc Research Ethics Committee (Ref. No. 27220) and the ‘Comité National d’Éthique pour la Recherche en Santé (CNERS)’ ethics committee in Senegal (Protocol SEN22/03). The study was introduced to and approved by key public health officials in Pikine, the area of study and the Department of Neglected Tropical Diseases at the Ministry of Health and Social Action of the Republic of Senegal. Written informed consent was obtained for each subject or from their parent or legal guardian. Additional information regarding the ethical, cultural, and scientific considerations specific to inclusivity in global research is included in the Supporting Information (S1 Checklist).

## 3. Results

**Fig 1:**
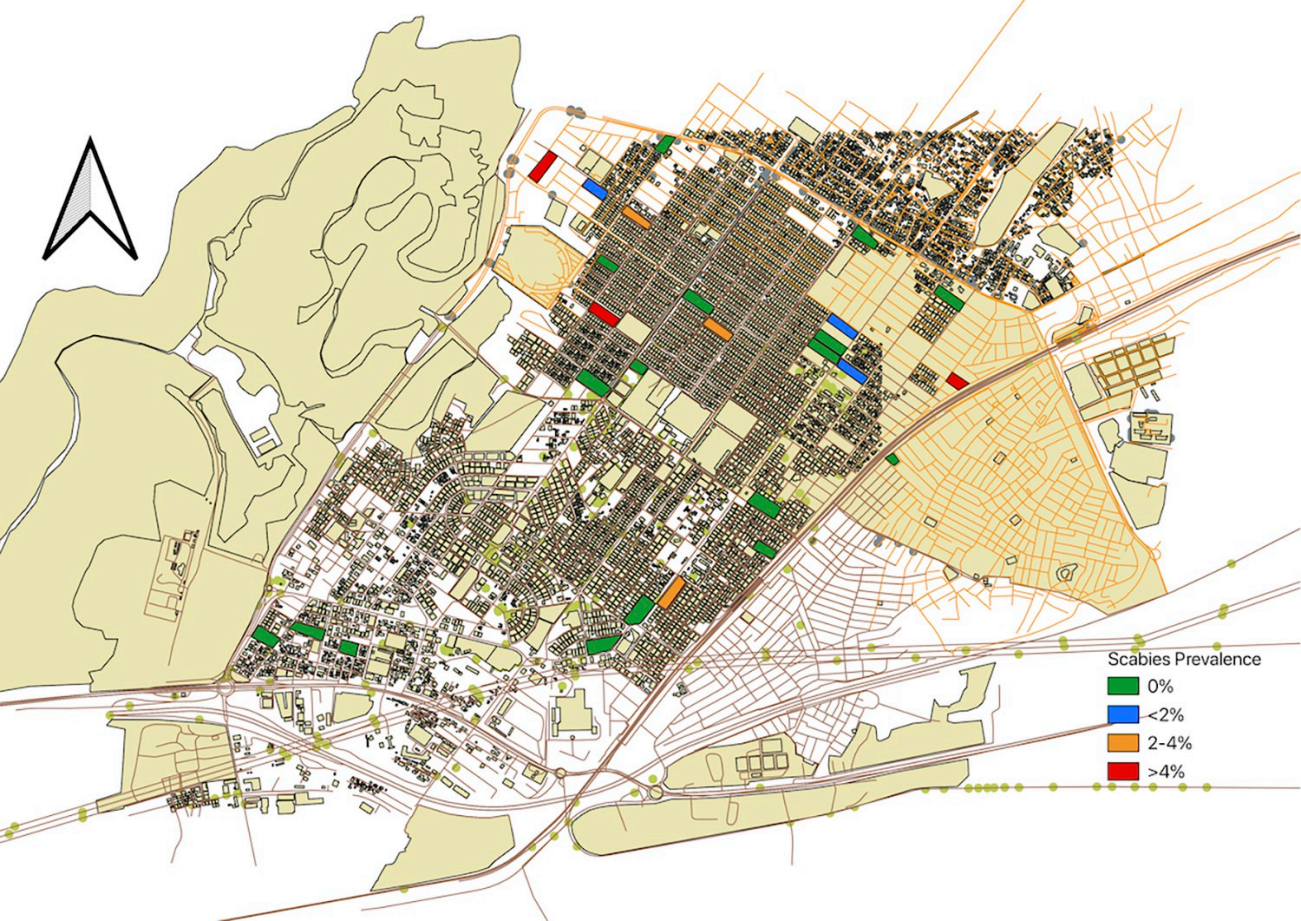
Map of Pikine, a suburb of Dakar, the most Western point of Africa, showing the 27 randomly selected clusters, color-coded for different levels of scabies prevalence in the respective clusters, from which participants were recruited: Green: No scabies, blue: >0–2% scabies, yellow: >2–4% scabies, red: >4–8.5% scabies. Map created in QGIS by the authors. The basemap is a custom download from OpenStreetMap (OSM). OSM is accessible under a CC-BY-SA-licence.

**Fig 2:**
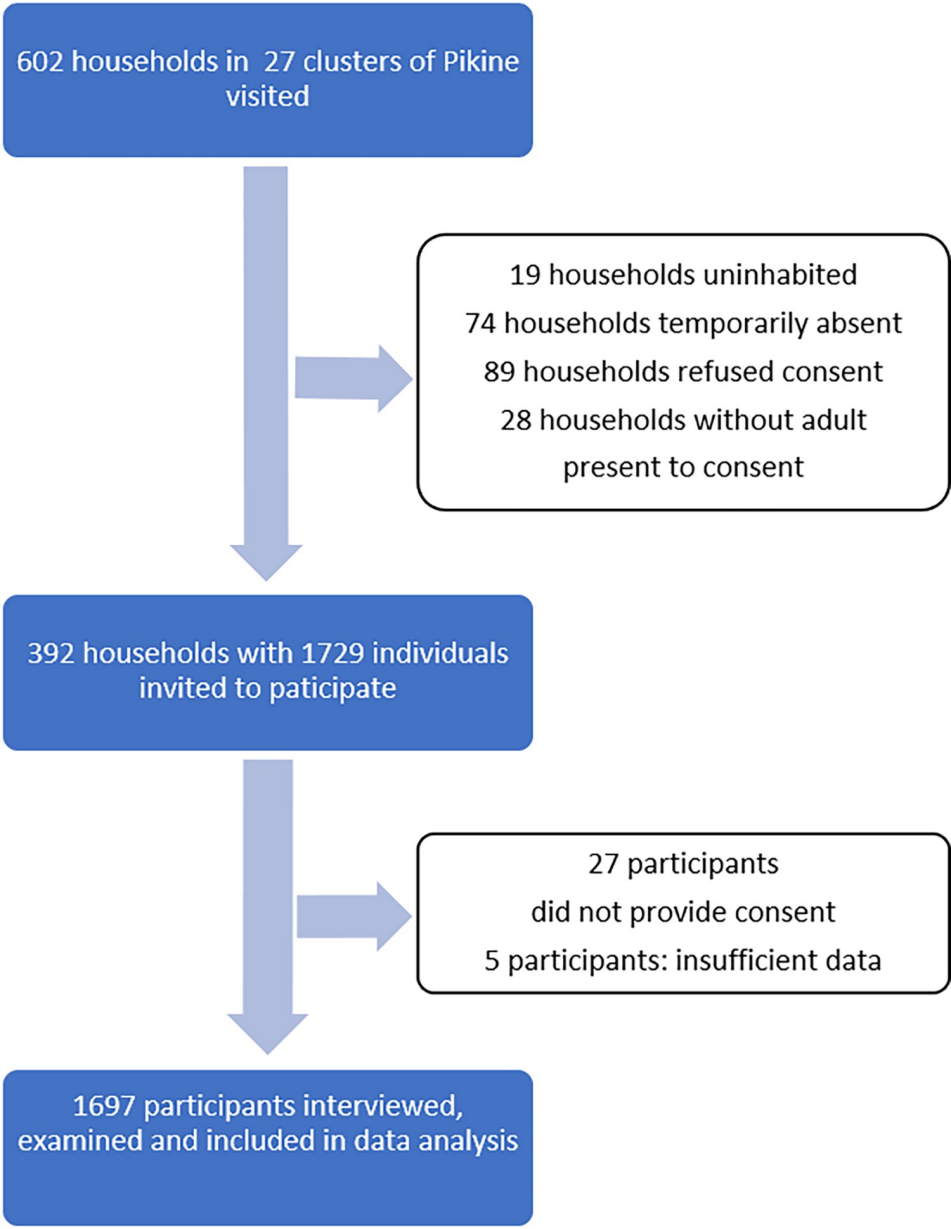
Flow chart of recruitment for scabies and impetigo survey in Pikine, a suburb of Dakar, Senegal.

Between July 19^th^ and August 5^th^ 2022, 1697 participants from 392 households were enrolled (see Figs 1 and 2). The study period fell in the national school holidays and at the start of the rainy season.

1207 (71.1%) participants were female. There was a broadly equal distribution of male and female participants in early childhood but a skewed distribution towards female participants was observed in working-age adults. The median age of the participants was 19 years (IQR 8–37 years). The majority of the population were Wolof, Fulani and Serer. The educational attainment for the head of households was generally low with 47.8% not having received any formal education, and of the remaining 36.9% had not completed secondary school (Table 1).

**Table 1:** Household size, education level, ethnicity of households and age, gender and place of education of individual participants (N = 1697).

Household Characteristics		
		N (%)
**Median Household Size** **N = 367 (IQR)**		**6 (4, 9)**
**Education of head of household (N=358)**	**No formal education**	**171 (47.8)**
	**School education up to partial secondary**	**132 (36.9)**
	**Secondary school education or higher**	**55 (15.4)**
**Ethnicity of Head of Household (N=387)**	**Wolof**	**155 (40.1)**
	**Fulani**	**78 (20.2)**
	**Serer**	**57 (14.7)**
	**Other**	**97 (25.1)**
**Participant Data**		
	**Category**	**N (%)**
**Gender (N = 1697)**	**Male**	**490 (28.9)**
	**Female**	**1207 (71.1)**
**Age group (N = 1697)**	**Age 0 to <5 yrs.**	**258 (15.2)**
	**Age 5 to <10 yrs.**	**222 (13.1)**
	**Age 10 to <15 yrs.**	**209 (12.3)**
	**Age 15 to <20 yrs.**	**178 (10.5)**
	**Age >20 yrs.**	**830 (48.9)**
**Current place of education of participants who were enrolled as students (N = 588)**	**Non-Koranic School**	**539 (91.7)**
	**Koranic School**	**49 (8.3)**

Overall, 27 people (1.6% 95% CI 0.8–3.2%) were diagnosed with scabies. Of these 26 were diagnosed as clinical scabies and one as suspected scabies (C1, according to the the 2020 IACS’s criteria). Amongst the male population, the scabies prevalence was 2.8% compared to 1.1% in females (adjusted odds ratio (OR) 3.1, 95% CI 1.4–6.8, p <0.01). The prevalence of scabies between the 27 clusters ranged from 0% to 8.5%. Amongst the different age categories, scabies was most prevalent amongst school-aged children between the ages of 5 and 10 years (4.5%) compared to 0.4% in the adult population. (Table 2). Of the 27 cases of scabies 11 were mild (40.7%), 3 were of moderate severity (11.1%) and 13 were severe (48.1%).

**Table 2:** Scabies and impetigo prevalence by age category, gender, school type and education of head of household.

	Participants No (%)	Cases with scabies N (%)	Adjusted* odds ratio (95% CI)**	p-value	Cases with impetigo No (%)	Adjusted* odds ratio (95% CI)**	p-value
**Age 0-<5 yrs.**	258 (15.2)	6 (2.3)	6.7 (1.6–27.5)	<0.01	3 (1.2)	9.9 (1.0–96.3)	0.05
**Age 5-<10 yrs.**	222 (13.1)	10 (4.5)	12.1 (3.2–45.4)	<0.01	4 (1.8)	14.2 (1.6–129.7)	0.02
**Age 10 to <15 yrs.**	209 (12.3)	7 (3.3)	9.1 (2.3–36.4)	<0.01	2 (1.0)	7.8 (0.7–87.8)	0.10
**Age 15 to <20 yrs.**	178 (10.4)	1 (0.6)	1.5 (0.1–14.4)	0.74	0 (-)	-	-
**Age >20 yrs.**	830 (48.9)	3 (0.4)	1	-	1 (0)	1	-
**Total**	**1697**	**27 (1.6)**	**(0.8–3.2)**	**-**	**10 (0.6)**	**(0.3–1.3)**	**-**
**Male**	490 (28.9)	14 (2.8)	3.1 (1.4–6.8)	<0.01	9 (1.8)	25 (3.1–200.5)	<0.01
**Female**	1207 (71.1)	13 (1.1)	1	-	1 (0%)	1	-
**Participant attending Koranic school**	49 (2.9)	10 (20.4)	17.6 (6.0–52.1)	<0.01	3	16.8 (2.4–118.5)	<0.01
**Participant attending regular school**	539 (31.8)	8 (1.5)	1	-	2	1	-
**Household head with any education**	701 (41.3)	7 (1.0)	0.5 (0.2–1.3)	0.16	2 (0.3)	0.61 (0.1–3.3)	0.57
**Household head without education**	702 (41.4)	18 (2.6)	1	-	6 (0.9)	1	-

*adjusted for age category, gender and school type.

** using logistic regression analysis corrected for cluster sampling design.

A total of 10 participants were diagnosed with impetigo (0.6%, 95% CI 0.3–1.3%). Of these, 5 also had scabies. The severity of impetigo was classified as very mild in 5 cases and mild in the remaining 5 cases. Overall, 9 of the 10 cases (90%) of impetigo were diagnosed in boys.

The prevalence of scabies was higher amongst children attending Koranic schools compared to non-Koranic schools (20.4% vs 1.5%, OR 17.6 95% CI 6.0–52.1, p <0.01). There was no significant association between the education of household heads and the presence of scabies or impetigo in participants (Table 2). Household size was also not associated with scabies.

For participants with scabies or impetigo who completed a DLQI quality-of-life questionnaire, the median score was 10.00 (IQR 8–12), for participants who completed a CDLQI the median score was 8.75 (IQR 4.5–13) and for participants where a FDLQI score was completed the median was 5.83 (IQR 2–10). This indicates a mild to moderate impact of the disease on the participants quality of life. With the total number of participants suffering from scabies or impetigo being very low (26 cases), we did not stratify the quality-of-life by disease severity.

## 4. Discussion

In this study, we found a relatively low prevalence of scabies and impetigo in a peri-urban region of Dakar. Cases of scabies were not evenly distributed in the population and were much more common in school-aged male children, in particular those who attended Koranic schools. This finding suggests that transmission of scabies in the general population is relatively limited and that most cases occur from transmission within specific facilities or locations such as schools, e.g. Koranic.

The prevalence of scabies seen in this study are lower than that reported in a survey in Monrovia in Liberia and a second survey in Sukuta in The Gambia [20,29]. Both of these surveys had a prevalence of scabies that approached 10% in the general population, suggesting that in these settings there was a more generalized transmission of scabies. In the absence of any recent health intervention to reduce the burden of disease in Pikine, there are several possible reasons for this difference in scabies prevalence. First, the GDP per capita in Senegal is about twice that of The Gambia and three times as high compared to Liberia. With poverty being a well-known risk factor for acquiring scabies, the higher GDP in Senegal might be an important reason, why we found a much lower prevalence in Senegal compared to neighboring countries. Second, scabies is associated with crowding, and whilst we have no information on the population density in the different locations, it is conceivable that with increased GDP living conditions in Pikine are less crowded compared to other urban survey sites. Third, it may be that the population in Pikine has better access to preventive or therapeutic health services than inhabitants in Monrovia or Sukuta. In addition, there may be other unknown geographic, seasonal, socio-economic, behavioral or public health reasons for the low burden of disease, which we were unable to explore.

We found that the health-related quality-of-life impact of scabies on peoples’ lives was mild to moderate which is broadly in line with previous studies [8,37,38]. As the total number of cases of scabies was low, our ability to assess relationships between disease severity and quality-of-life scores was limited.

Compared to the overall study population we found a very high rate of scabies infection in pupils attending Koranic schools compared to attendants of non-Koranic schools. As a result, individuals attending Koranic schools made up more than one third of all cases with scabies despite representing less than 3% of participants. We have previously shown a high prevalence of scabies amongst children at Koranic schools. Studies in Dakar and from Asian countries have found that problems related to hygiene and crowding were risk factors for the high transmission of scabies in these institutions [30] The increased rate of scabies in male survey participants likely reflects this increased burden amongst attendees at Koranic schools. Our data suggest targeted interventions in these institutions may be the most effective strategy to reduce the burden of scabies in Pikine.

Our study has a number of strengths including a large sample size, the use of a robust cluster-randomized sampling approach and the use of standardized examinations using the IACS criteria performed by well trained staff. The entire period of recruitment fell into the school summer holidays in Senegal, which allowed us to recruit a large proportion of the school-aged population that had a high prevalence of scabies. This would be important to consider for other population-based surveys as well. This survey also has a number of limitations. Given the nature of the fieldwork, we did not perform dermatoscopy or other direct diagnostic visualisation methods. However, the performance of the clinical IACS criteria has previously been shown to be accurate at the population level, and we do not think this will have impacted our findings. We undertook a cross-sectional survey and cannot exclude that seasonal variations might occur, as has been shown for impetigo in The Gambia [29]. Despite best efforts to enroll all members of each household, recruitment took place during working hours only, resulting in an under representation of adult men, which has also been seen in other studies [20].

In summary, we found a relatively low overall prevalence of scabies in the general population but evidence of much higher levels of transmission in particular environments, notably Koranic schools. Our data highlight the need for scabies prevalence surveys to consider not only the overall population prevalence but also specific sub-populations where targeted interventions may be required. Further studies specifically involving Koranic schools, including intervention studies, should be considered.

## Supporting information

S1 Checklist Inclusivity in global research. (DOCX)

S1 Data Data file. (CSV)
